# The Arctic Plant Aboveground Biomass Synthesis Dataset

**DOI:** 10.1038/s41597-024-03139-w

**Published:** 2024-03-20

**Authors:** Logan T. Berner, Kathleen M. Orndahl, Melissa Rose, Mikkel Tamstorf, Marie F. Arndal, Heather D. Alexander, Elyn R. Humphreys, Michael M. Loranty, Sarah M. Ludwig, Johanna Nyman, Sari Juutinen, Mika Aurela, Konsta Happonen, Juha Mikola, Michelle C. Mack, Mathew R. Vankoughnett, Colleen M. Iversen, Verity G. Salmon, Dedi Yang, Jitendra Kumar, Paul Grogan, Ryan K. Danby, Neal A. Scott, Johan Olofsson, Matthias B. Siewert, Lucas Deschamps, Esther Lévesque, Vincent Maire, Amélie Morneault, Gilles Gauthier, Charles Gignac, Stéphane Boudreau, Anna Gaspard, Alexander Kholodov, M. Syndonia Bret-Harte, Heather E. Greaves, Donald Walker, Fiona M. Gregory, Anders Michelsen, Timo Kumpula, Miguel Villoslada, Henni Ylänne, Miska Luoto, Tarmo Virtanen, Bruce C. Forbes, Norbert Hölzel, Howard Epstein, Ramona J. Heim, Andrew Bunn, Robert M. Holmes, Jacqueline K. Y. Hung, Susan M. Natali, Anna-Maria Virkkala, Scott J. Goetz

**Affiliations:** 1 School of Informatics, Computing, and Cyber Systems, Northern Arizona University, Flagstaff, USA; 2 Department of Ecoscience, Aarhus University, Aarhus, Denmark; 3 College of Forestry, Wildlife, and Environment, Auburn University, Auburn, USA; 4 Department of Geography and Environmental Studies, Carleton University, Ottawa, Canada; 5 Department of Geography, Colgate University, Hamilton, USA; 6 Department of Earth and Environmental Sciences, Columbia University, Palisades, USA; 7 Jeb E. Brooks School of Public Policy, Cornell University, Ithaca, USA; 8 Climate System Research, Finnish Meteorological Institute, Helsinki, Finland; 9 Finnish Meteorological Institute, Helsinki, Finland; 10 Finnish Youth Research Society, Helsinki, Finland; 11 Bioeconomy and Environment Unit, Natural Resources Institute Finland, Helsinki, Finland; 12 Center for Ecosystem Science and Society, Northern Arizona University, Flagstaff, USA; 13 Department of Biological Sciences, Northern Arizona University, Flagstaff, USA; 14 Applied Research, Nova Scotia Community College, Middleton, Canada; 15 Climate Change Science Institute, Oak Ridge National Laboratory, Oak Ridge, USA; 16 Environmental Science Division, Oak Ridge National Laboratory, Oak Ridge, USA; 17 Department of Biology, Queen’s University, Kingston, Canada; 18 Department of Geography and Planning, Queen’s University, Kingston, Canada; 19 Department of Ecology and Environmental Science, Umeå University, Umeå, Sweden; 20 Département des sciences de l’environnement, Université du Québec à Trois-Rivières, Trois-Rivières, Canada; 21 Centre d’Études Nordiques, Université Laval, Québec, Canada; 22 Department of Biology, Université Laval, Québec, Canada; 23 Department of Plant Science, Université Laval, Québec, Canada; 24 Geophysical Institute, University of Alaska Fairbanks, Fairbanks, USA; 25 Institute of Arctic Biology, University of Alaska Fairbanks, Fairbanks, USA; 26 Alberta Biodiversity Monitoring Institute, University of Alberta, Edmonton, Canada; 27 Department of Biology, University of Copenhagen, København, Denmark; 28 Department of Geographical and Historical Studies, University of Eastern Finland, Joensuu, Finland; 29 Institute of Agriculture and Environmental Sciences, Estonian University of Life Sciences, Tartu, Estonia; 30 School of Forest Sciences, University of Eastern Finland, Joensuu, Finland; 31 Department of Geosciences and Geography, University of Helsinki, Helsinki, Finland; 32 Ecosystems and Environment Research Program, University of Helsinki, Helsinki, Finland; 33 Arctic Centre, University of Lapland, Rovaniemi, Finland; 34 Institute of Landscape Ecology, University of Münster, Münster, Germany; 35 Department of Environmental Science, University of Virginia, Charlottesville, USA; 36 Department of Evolutionary Biology and Environmental Studies, University of Zurich, Zürich, Switzerland; 37 Department of Environmental Sciences, Western Washington University, Bellingham, USA; 38 Woodwell Climate Research Center, Falmouth, USA

**Keywords:** Ecosystem ecology, Databases, Plant ecology, Carbon cycle, Ecological modelling

## Abstract

Plant biomass is a fundamental ecosystem attribute that is sensitive to rapid climatic changes occurring in the Arctic. Nevertheless, measuring plant biomass in the Arctic is logistically challenging and resource intensive. Lack of accessible field data hinders efforts to understand the amount, composition, distribution, and changes in plant biomass in these northern ecosystems. Here, we present *The Arctic plant aboveground biomass synthesis dataset*, which includes field measurements of lichen, bryophyte, herb, shrub, and/or tree aboveground biomass (g m^−2^) on 2,327 sample plots from 636 field sites in seven countries. We created the synthesis dataset by assembling and harmonizing 32 individual datasets. Aboveground biomass was primarily quantified by harvesting sample plots during mid- to late-summer, though tree and often tall shrub biomass were quantified using surveys and allometric models. Each biomass measurement is associated with metadata including sample date, location, method, data source, and other information. This unique dataset can be leveraged to monitor, map, and model plant biomass across the rapidly warming Arctic.

## Background & Summary

Plant biomass not only shapes how humans and wildlife use terrestrial ecosystems^1–3^ but also influences climate by modulating ecosystem carbon storage and surface energy balance^4–6^. However, plant biomass and its associated ecosystem services are sensitive to rapid climate warming, which is occurring at least three times faster in the Arctic than anywhere else on the planet^7,8^. Rapid warming of Arctic ecosystems has enabled plants such as shrubs and trees to grow taller and expand across the land surface^9–12^ and vegetation to become more productive^13–17^. These changes can affect traditional land use^18,19^, impact habitat suitability for wildlife^3,15,20^, and amplify climate warming, primarily by lowering the surface albedo^4,6^. Consequently, there is a pressing need to better understand spatial patterns and temporal changes in plant biomass and species distribution throughout Arctic ecosystems.

Field measurements are crucial for quantifying the amount, composition, distribution, and temporal changes in plant biomass across Arctic ecosystems. While recognizing the importance of efforts like The International Tundra Warming Experiment^21^ and US National Ecological Observatory Network^22^, it is nevertheless uncommon for plant biomass to be systematically measured and monitored in Arctic ecosystems, where such measurements are time-consuming, logistically challenging, and resource intensive. Rather, plant biomass typically has been measured as part of individual research projects, each with its own focus and protocols. For instance, researchers have investigated how plant biomass is affected by climate^23,24^, wildfire^25–28^, herbivores^29,30^, and soil properties such as texture, nutrients, and pH^31–33^. Researchers have also measured plant biomass to assess ecosystem carbon storage^34,35^, evaluate terrestrial ecosystem models^36^, and map spatial patterns of plant biomass from landscape to biome scales by linking field and remote sensing measurements^37–40^.

Researchers typically measure plant aboveground biomass by harvesting sample plots during mid- to late-summer, though measuring tall shrub and tree biomass generally requires surveying stems on sample plots and using allometric models^34,41^. However, the number and size of sample plots varies among research projects, as do the taxonomic and functional groupings used when partitioning samples. Samples are sometimes partitioned by species, or more coarsely partitioned into plant functional types that include multiple species with similar functional traits^42^. Furthermore, while researchers are progressively archiving individual datasets in a growing number of online public repositories, finding datasets can be challenging and many datasets remain unarchived. Even when archived, it is still necessary to harmonize datasets before they can be used together to inform larger-scale biomass monitoring and mapping efforts. So far, there have been limited efforts to compile and harmonize plot-level measurements of plant aboveground biomass across individual datasets, either regionally^43,44^ or for the overall Arctic^37,45^. Altogether, these factors hinder efforts to understand spatial patterns and temporal changes in plant biomass across the rapidly warming Arctic.

**Fig. 1:**
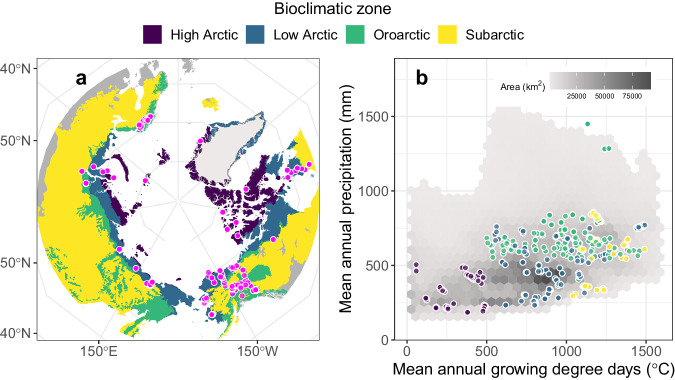
Synthesis dataset field site locations in (**a**) geographic and (**b**) climatic spaces. The synthesis dataset includes field sites from the sparsely vegetated High Arctic, moderately vegetated Low Arctic, mountainous Oroarctic, and forested Subarctic. (**a**) Bioclimatic zones were derived from several datasets^98–100^ and clipped to north of 55°N. (**b**) Climatologies are for the period 1981 to 2010 based on the CHELSA dataset gridded at 1 km^2^ resolution (version 2.1)^101,102^. Growing degree days represent the heat sum above 0 °C. To improve clarity, panel (**b**) excludes the Subarctic, the warmest and wettest 2.5^th^ percentiles, and climate spaces (i.e., unique growing degree day and precipitation combinations) that covered less than 500 km^2^.

**Fig. 2:**
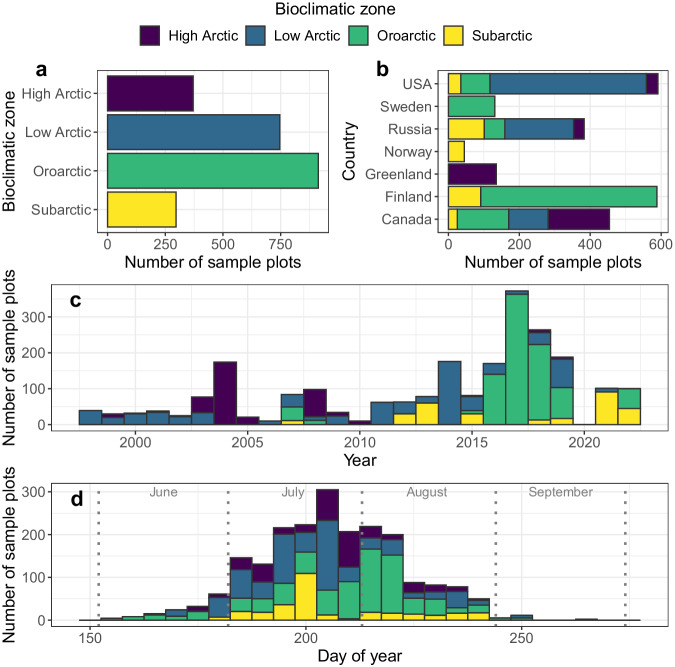
Frequency distributions of where and when sample plots were measured. Specifically, the (**a**) bioclimatic zone, (**b**) country, (**c**) year, and (**d**) day of year in which sample plots were measured. (**b,c,d**) Histogram bars are subdivided and color-coded by bioclimatic zones.

Here, we present *The Arctic plant aboveground biomass synthesis dataset*, which includes georeferenced measurements of lichen, bryophyte, herb (graminoid and forb), shrub, and/or tree aboveground biomass on 2,327 sample plots from 636 field sites across Arctic and Subarctic ecosystems (Fig. 1). These five plant functional types correspond to broad differences in trait characteristics (e.g., height, woodiness, vascularity) and effects on ecosystem processes^42^ (Table 1), and are commonly used by terrestrial ecosystem models to represent plant form and function^46,47^. We created the synthesis dataset by assembling and harmonizing 32 datasets where aboveground biomass was quantified by harvesting sample plots, or, for trees and often tall shrubs, by surveying sample plots and using allometric models. Aboveground biomass is reported for each plant functional type as grams of oven-dried aboveground live biomass per square meter of ground surface (g m^−2^), and in most cases represents the peak summer biomass on each sample plot. The synthesis dataset does not include measurements of belowground biomass, which were recently compiled elsewhere^45^, or biomass chemistry (e.g., carbon or nitrogen content). Altogether, the synthesis dataset includes measurements that span a broad range of bioclimatic conditions across seven of the eight Arctic nations (Figs. 1, 2a,b). The synthesis dataset can be used for a variety of ecological applications that include monitoring, mapping, and modeling spatial patterns and temporal changes in plant aboveground biomass across the Arctic.

**Table 1:** Description of plant functional types used in *The Arctic plant aboveground biomass synthesis dataset*.

Name	Brief description	Examples
Lichen	Fungus engaged in mutualistic symbiosis with photosynthesizing algae and/or cyanobacteria.	Reindeer lichen (*Cladonia rangiferina*), leafy lichen (*Peltigera aphthosa*), curled snow lichen (*Flavocetraria cucullata*)
Bryophyte	Non-vascular plants including mosses, liverworts, and hornworts.	Rusty bogmoss *(Sphagnum fuscum)*, ribbed bogmoss *(Aulacomnium palustre)*
Herb	Vascular non-woody plants including sedges, grasses, and forbs.	Tussock cottongrass (*Eriophorum vaginatum*), water sedge (*Carex aquatilis*), alpine bluegrass (*Poa alpina*), horsetail (*Equisetum arvense*), fireweed (*Chamerion angustifolium*)
Shrub	Multi-stem vascular woody plants with deciduous or evergreen leaves.	Dwarf birch (*Betula nana*), Siberian alder (*Alnus viridis*), crowberry (*Empetrum nigrum*)
Tree	Single-stem vascular woody plants with deciduous or evergreen leaves.	White spruce (*Picea glauca*), Cajander larch (*Larix cajanderi*), Mountain birch (*Betula pubescens*)

Plant functional types were adapted from Chapin *et al*.^40^. Although lichen biomass is predominantly fungal^41^ rather than algal or cyanobacterial, the symbiosis is included here as a plant functional type.

## Methods

### General approach

**Fig. 3:**
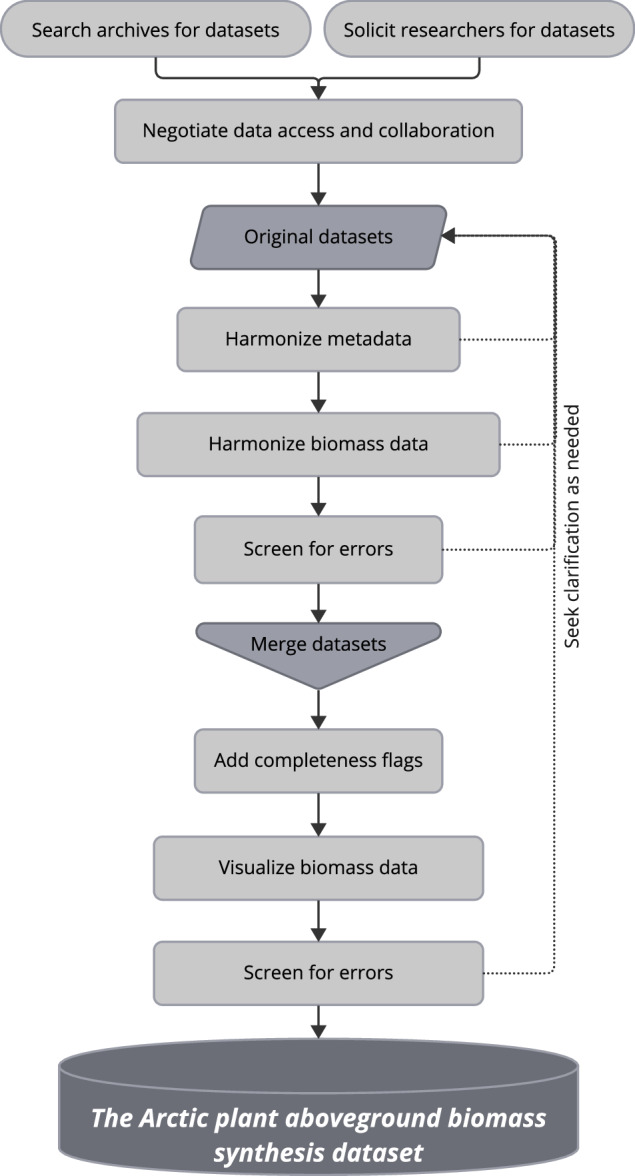
Workflow diagram depicting the process for creating *The Arctic plant aboveground biomass synthesis dataset* from existing datasets. Harmonization of metadata and biomass data included reformatting sample dates and spatial coordinates into common formats, as well as summarizing aboveground biomass by a common set of plant functional types.

To create the synthesis dataset, we assembled, harmonized, and screened individual datasets. We then merged the harmonized datasets, added completeness flags, and performed further quality assurance. The workflow is depicted in Fig. 3, with further details provided below.

### Dataset sources

We assembled datasets by searching public data archives and directly soliciting datasets from the authors of relevant scientific papers and members of our professional networks. We searched public data archives and Google Scholar using combinations of keywords that included *Arctic*, *tundra*, *vegetation*, *plant*, and *biomass*. Data archives included the Arctic Data Center, DataOne, Dryad, Oak Ridge National Laboratory Distributed Active Archive Center for Biogeochemical Dynamics, PANGAEA Data Publisher for Earth and Environmental Science, Polar Data Catalog, and Zenodo. After identifying datasets and performing an initial screening, we contacted the researchers who created the dataset, sought additional information as needed, requested permission to include the dataset in the synthesis, and invited those researchers to be coauthors on the synthesis dataset. In total, we assembled 32 individual datasets provided by 54 researchers at 28 institutions in eight countries (Table 2).

**Table 2:** Summary of individual datasets that comprise *The Arctic plant aboveground biomass synthesis dataset*.

Data source	Country	Lat.	Lon.	Plots	Years
Danby, *et al*.^77^	Canada	61.24	−138.52	50	2007–2008
Deschamps, *et al*.^30^	Canada	73.15	−79.95	8	2018
Gaspard and Boudreau^55^	Canada	59.43	−75.52	52	2018–2021
Gignac, *et al*.^78^	Canada	73.16	−80.02	6	2019
Gregory^53^	Canada	74.91	−109.58	66	2008
Hayne^79^	Canada	64.87	−111.57	24	2009
Lafleur and Humphreys^80^	Canada	64.87	−111.57	10	2006
Lafleur and Humphreys^81^	Canada	64.87	−111.58	15	2007–2011
Skaarup^82^	Canada	64.87	−111.59	9	2015–2015
Vankoughnett and Grogan^83^	Canada	64.87	−111.56	10	2008
Orndahl^84^	Canada/USA	65.64	–141.92	214	2018–2019
Walker, *et al*.^51^	Canada/USA	72.39	−134.02	209	2000–2005
Berner, *et al*.^85^	Finland	69.45	25.25	100	2022
Happonen, *et al*.^68^	Finland	69.06	20.82	442	2016–2018
Villoslada, *et al*.^29^	Finland/Norway	68.83	23.88	91	2021
Arndal, *et al*.^86^	Greenland	74.48	−20.53	135	2004
Heard, *et al*.^87^	Russia	68.88	161.44	117	2012–2013
Heim, *et al*.^25^	Russia	67.01	79.14	58	2017–2018
Loranty and Natali^88^	Russia	69.32	161.56	26	2014
Mikola, *et al*.^89^	Russia	71.59	128.89	92	2014
Siewert, *et al*.^35^	Russia	70.83	147.47	24	2012
Walker, *et al*.^23^	Russia	71.23	67.64	66	2007–2010
Siewert and Olofsson^38^	Sweden	68.37	18.52	131	2017
Bret-Harte, *et al*.^90^	USA	68.94	−150.24	19	2011
Bret-Harte, *et al*.^91^	USA	68.95	−150.21	19	2011
Bret-Harte, *et al*.^92^	USA	69.00	−150.29	19	2011
Greaves, *et al*.^93^	USA	68.63	−149.58	58	2014
Hung, *et al*.^94^	USA	61.27	−163.21	54	2018–2019
Ludwig, *et al*.^95^	USA	61.26	−163.00	27	2016–2017
Natali, *et al*.^96^	USA	66.54	−149.81	72	2015
Raynolds^97^	USA	68.53	−158.19	92	1998–2000
Salmon, *et al*.^50^	USA	65.16	−164.82	12	2016

The coordinates represent the average latitude and longitude of sample plots in each dataset.

### Metadata harmonization

We harmonized plot-level plant biomass measurements and metadata from individual datasets using custom scripts written in R^48^. These scripts provide a record of the harmonization process and enable future updates. For each dataset, we assigned a unique sequential identifier, recorded the names of data contributors, and included a citation to the original peer-reviewed paper or dataset, thereby enabling users to trace the origin of each measurement. For each unique sample plot in the dataset, we identified the country of origin, assigned a general locale, and recorded the original field site ID and sample plot ID. Site ID and plot ID may not be unique identifiers. Therefore, to ensure that each field site and sample plot could be uniquely identified in the synthesis dataset, we created site codes and plot codes by concatenating the country, locale, site ID, and plot ID. We documented whether the GPS coordinates were recorded at the site or plot level, then harmonized the coordinates to decimal degrees in the WGS84 global reference system using the *sf* package in R^49^.

The definition of a field site varied among individual datasets. Most often, a field site included multiple sample plots along one or more transects in a single vegetation type. In other cases, a field site included sample plots spread among multiple vegetation types in a landscape^29,35,38,50,51^. In these later cases, we subdivided the field site by grouping sample plots by vegetation types (e.g., low shrub tundra vs. graminoid tundra) that were recorded by the researchers who conducted the field work. This helped to ensure that in the synthesis dataset, each field site included multiple sample plots (i.e., replicate measurements) from a single vegetation type.

### Plant aboveground biomass measurement harmonization

Plant aboveground biomass was quantified for most functional types by harvesting sample plots during mid- to late-summer. Typically, non-tree vascular plants rooted in a sample plot were clipped at the moss or ground surface and sorted into functional types (e.g., herbs, shrubs) or finer taxonomic groupings (e.g., species). If present, lichen and the green portion of mosses and other bryophytes were then harvested. Samples were dried to a constant weight typically at 50–60 °C using a drying oven and weighed using a digital scale. Trees and tall shrubs are challenging to harvest and process; therefore, tree and often tall shrub aboveground biomass were quantified on sample plots by (1) measuring the diameter of each stem at the ground surface or chest height, (2) predicting stem dry weight from stem diameter using allometric models^41,52^, and (3) summing stem dry weight across all stems on the sample plot. For some sample plots, dwarf to low shrubs were harvested while tall shrubs were surveyed. The synthesis dataset includes the sampling date and quantification method for each plant biomass measurement.

Individual datasets differed in the taxonomic detail of plant biomass measurements. While some datasets provided measurements for individual species and one dataset provided measurements of total aboveground biomass^53^, most datasets instead provided measurements for species-groups or broader plant functional types. Therefore, it was necessary to aggregate the plant biomass measurements to a harmonized set of plant functional types, with the level of taxonomic detail dictated by the most coarsely partitioned datasets. The synthesis dataset therefore includes plant biomass measurements that were aggregated to five plant functional types: lichens, bryophytes, herbs, shrubs, and trees (Table 1). Lichens are predominantly fungal^54^, yet are often included as a plant functional type in Arctic ecology^42^.

Plant biomass is expressed as grams of oven-dried aboveground live biomass per square meter of ground surface (i.e., g m^−2^); however, the actual area of each sample plot widely varied among individual datasets and plant functional types. For instance, bryophytes and lichens are small and particularly time consuming to harvest, thus sample plots typically were about 0.1 m^2^. In several cases, bryophyte and lichen biomass were upscaled using targeted harvests and measurements of functional type cover on a larger sample plot^35,55^. Herbs and shrubs were typically harvested from sample plots that were about 0.25 m^2^, while tall shrub and tree biomass were quantified by surveying sample plots up to 25 m^2^ and 100 m^2^, respectively. The synthesis dataset therefore includes the area (m^2^) of the sample plot that was used when measuring plant biomass for each functional type.

We sought to assemble plant biomass measurements for all functional types present on each sample plot; however, there were cases when a plant functional type was present but not measured. This was most common for bryophytes and lichens. Several datasets were missing plant biomass measurements for certain functional types but had ancillary estimates of areal cover by functional type. We set plant biomass to 0 g m^−2^ for functional types that had 0% cover and added a note to document this decision. We took special care to document as “unmeasured” when a plant functional type was present in a sample plot but not measured (i.e., missing data). Therefore, every sample plot in the synthesis dataset includes a discrete biomass measurement or documented missing value for each of the five plant functional types. Furthermore, each sample plot has a set of logical flags (i.e., true or false) that identify which groups of plant functional types were measured (e.g., all vascular or woody functional types). These flags can help guide appropriate use of the synthesis dataset.

## Data Records

*The Arctic plant aboveground biomass synthesis dataset* is publicly available online through the Arctic Data Center^56^. The dataset includes one file in a comma-separated format (.csv) that has 11,372 rows and 33 columns. The first-row stores column names, while each subsequent row stores the biomass measurements and associated metadata for a single plant functional type (e.g., shrubs) on a sample plot. The dataset has 17 columns with character values, eleven columns with numeric values, and five columns with logical flags. Details about each column are provided in Table 3.

**Table 3:** Description of each column in *The Arctic plant aboveground biomass synthesis dataset*.

#	Column Name	Format	Units	Description
1	dataset_id	character	—	Dataset identifier assigned upon intake
2	contributor	character	—	Name(s) of data contributor(s)
3	country	character	—	Name of country where field data were collected
4	locale	character	—	Name of the general area where field data were collected
5	site_id	character	—	Site identifier that is unique within an individual dataset
6	site_code	character	—	Site code that is unique within the synthesis dataset based on concatenation of country, locale, and site_id
7	plot_id	character	—	Plot identifier that is unique within an individual site
8	plot_code	character	—	Plot code that is unique within the synthesis dataset based on concatenation of country, locale, site_id, and plot_id
9	coord_type	character	—	Coordinate type (*site* or *plot*)
10	latitude	numeric	°	Site or plot latitude in decimal degrees (WGS84)
11	longitude	numeric	°	Site or plot longitude in decimal degrees (WGS84)
12	year	numeric	year	Year when field data were collected
13	month	numeric	month	Month when field data were collected
14	day	numeric	day	Day of month when field data were collected
15	pft	character	–	Plant functional type (*lichen*, *bryophyte, herb*, *shrub*, *tree*, or *total*)
16	plot_area_m2	numeric	m^2^	Plot area used to harvest or survey vegetation
17	method	character	—	Method used to determine biomass dry weight (*harvest*, *survey*, *harvest + survey*, or *unmeasured*)
18	biomass_dry_weight_g	numeric	g	Aboveground biomass on sample plot in units of grams of oven-dried live biomass
19	biomass_density_gm2	numeric	g m^2^	Aboveground biomass on sample plot in units of grams of oven-dried live biomass per square meter of ground surface
20	vegetation_description	character	—	Description of the vegetation community where sampling occurred
21	site_description	character	—	Description of the field site where sampling occurred
22	bioclim_zone	character	—	Bioclimatic zone (*High Arctic*, *Low Arctic*, *Oroarctic*, or *Subarctic*)
23	mat_degC	numeric	°C	Mean annual temperature (1981–2010) from the Chelsa Bioclim dataset
24	gdd_degC	numeric	°C	Mean annual growing degree days (1981–2010) with a 0 °C base from the Chelsa Bioclim dataset
25	map_mm	numeric	mm	Mean annual precipitation (1981–2010) from the Chelsa Bioclim dataset
26	community_measured	logical	—	Were lichen, bryophyte, herb, shrub, and tree biomass measured on the sample plot? (*true* or *false*)
27	plants_measured	logical	—	Were bryophyte, herb, shrub, and tree biomass measured on the sample plot? (*true* or *false*)
28	nontree_measured	logical	–	Were lichen, bryophyte, herb, and shrub biomass measured on the sample plot? (*true* or *false*)
29	vascular_measured	logical	—	Were herb, shrub, and tree biomass measured on the sample plot? (*true* or *false*)
30	woody_measured	logical	—	Were shrub and tree biomass measured on the sample plot? (*true* or *false*)
31	citation	character	—	Full citation to the original data source
32	citation_short	character	—	Shortened citation to the original data source
33	notes	character	—	Miscellaneous notes

Altogether, the synthesis dataset requires about 7 MB of hard drive storage space.

## Technical Validation

We took multiple steps to ensure the technical quality of *The Arctic plant aboveground biomass synthesis dataset*. For individual datasets (n = 32), we started by examining the structure of the tabular data, as well as visually screening these data for potential errors (e.g., typographical errors). Individual datasets were unique; therefore, we harmonized each dataset using a custom script in R^48^. These scripts provide documented and refinable workflows for data harmonization, which included, but were not limited to, fixing typographical errors, and screening the spatial coordinates for each field site and/or sample plot. Specifically, we visually screened spatial coordinates for irregularities by mapping each reported location over high-resolution satellite imagery using the R package *leaflet*^57^. Accurate spatial coordinates are especially important for ecosystem monitoring and mapping. Each script also included checks to ensure there were plot-level data for all five plant functional types and, after harmonization, that the dataset columns matched the synthesis dataset.

**Fig. 4:**
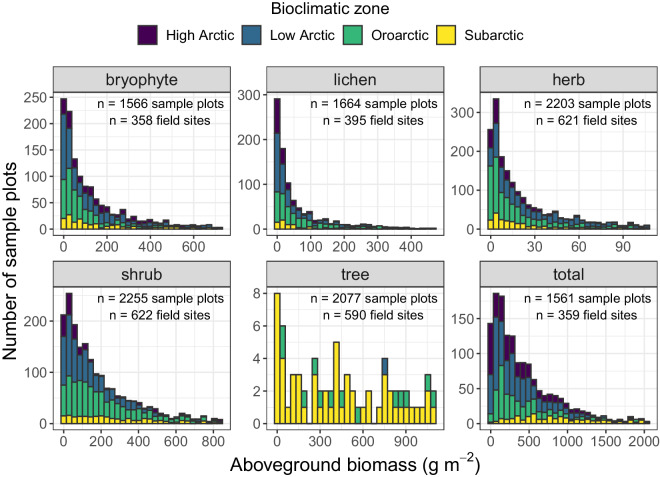
Frequency distribution of plant aboveground biomass (g m^−2^) by functional type for sample plots in *The Arctic plant aboveground biomass synthesis dataset*. To improve clarity, (1) the x-axis is limited to 95% of the maximum range in aboveground biomass for each plant functional type, and (2) sample plots are not shown if there was no biomass (i.e., 0 g m^−2^) for the plant functional type. The total number of sample plots and field sites with biomass measurements is provided for each plant functional type.

We created the synthesis dataset by merging the individual harmonized datasets and then performed additional screening using R. To ensure data quality for each column with character values, we extracted the unique values and visually checked for errors. For each column with numeric values, we calculated the range of values and similarly checked for errors. Plant aboveground biomass (g m^−2^) is the principal measurement in the synthesis dataset; therefore, we further examined these numeric values. This included visually inspecting histograms for each plant functional type (Fig. 4), as well as computing standardized anomalies (i.e., z-scores) and inspecting measurements with anomalies greater than three standard deviations for errors.

To further validate the synthesis dataset, we compared the range of total aboveground biomass values in the synthesis dataset with values reported by several prior syntheses^43,58^ and found they were of similar magnitudes. Gilmanov and Oechel^43^ reported that total aboveground biomass ranged from 3 g m^−2^ to 4,058 g m^−2^ among 56 field sites in Subarctic and Arctic ecosystems across North America and Greenland. In our synthesis dataset, total aboveground biomass ranged from 2 g m^−2^ to 3,123 g m^−2^ among 182 field sites in the same regions, excluding two forest sites in the Subarctic with 6,261 and 8,947 g m^−2^. Similarly, Wielgolaski^58^ reported that total non-tree aboveground biomass ranged from 57 g m^−2^ to 2,162 g m^−2^ across 14 field sites in Subarctic and Arctic ecosystems in the USSR and Norway^44^. In our synthesis dataset, total non-tree aboveground biomass ranged from 15 g m^−2^ to 2,344 g m^−2^ across 60 field sites in the same regions, excluding one site in a dense riparian shrub thicket with 8,218 g m^−2^. In our entire synthesis dataset, only 2.5% of field sites had total aboveground biomass greater than 4,000 g m^−2^ (max = 8,947 g m^−2^), almost all of which were Subarctic forests. Total aboveground biomass tends to be much lower in Arctic tundra than Subarctic forests, where total aboveground biomass averages ~6,000 g m^−2^ but can range from ~2,000 g m^−2^ to ~30,000 g m^−2^ depending on climate and disturbance history^59,60^.

We further examined how aboveground biomass varied for plant functional types both within and across bioclimatic zones (Table 4) as compared with previously reported patterns. However, it is important to recognize that field sites in our synthesis dataset are not random or stratified samples of these bioclimatic zones and thus summary statistics may be biased. Nevertheless, the most pronounced pattern was an increase in median shrub aboveground biomass from ~35 g m^−2^ in the High Arctic to ~140 g m^−2^ in the Low Arctic, reaching ~190 g m^−2^ in Oroarctic and ~340 g m^−2^ in the Subarctic. Similarly, the median total aboveground biomass increased from ~340 g m^−2^ in the High Arctic to 1,230 g m^−2^ in the Subarctic. General increases in shrub and total aboveground biomass from the High Arctic to the Subarctic are well-documented macroecological patterns^23,24^. Also consistent with prior research^23^, we observed that median bryophyte and shrub aboveground biomass were consistently higher than median lichen, herb, or tree aboveground biomass, with bryophytes comprising the largest proportion of total aboveground biomass in the High Arctic and shrubs the largest proportion in the Low Arctic. However, it is important to note there is high spatial variability in the amount and composition of plant aboveground biomass among field sites in each bioclimatic zone, reflecting pronounced heterogeneity within and among vegetation communities^61,62^.

**Table 4:** Summary of aboveground biomass (g m^−2^) by plant functional type for each bioclimatic zone.

Plant Functional Type	Bioclimatic Zone			
	High Arctic	Low Arctic	Oroarctic	Subarctic
bryophyte	115 (21–275, n = 43)	118 (23–241, n = 122)	49 (24–95, n = 115)	90 (20–327, n = 78)
lichen	7 (1–29, n = 43)	10 (0–66, n = 131)	21 (0–71, n = 130)	5 (0–98, n = 91)
herb	10 (4–29, n = 43)	24 (11–52, n = 144)	6 (1–23, n = 340)	12 (4–30, n = 94)
shrub	35 (2–90, n = 43)	140 (52–300, n = 147)	192 (84–334, n = 340)	342 (182–562, n = 92)
tree	0 (0-0, n = 43)	0 (0-0, n = 145)	0 (0-0, n = 317)	11 (0–1474, n = 85)
*total*	338 (140–694, n = 55)	443 (224–800, n = 120)	429 (180–876, n = 94)	1,228 (911–2,010, n = 65)

Metrics include median aboveground biomass and, in parentheses, the interquartile range (i.e., 25th to 75th percentiles) and number of field sites. Biomass was first averaged across sample plots within each field site and then summarized across field sites within each bioclimatic zone. Total aboveground biomass was only computed when all five plant functional types were measured on a sample plot.

## Usage Notes

It is important to be aware of potential uncertainties and limitations when using the synthesis dataset, including uncertainties related to quantifying plant aboveground biomass on sample plots. First, it can be challenging to establish sample plot boundaries and identify which plants are rooted inside the plot and spreading outside the plot, versus rooted outside and spreading in. Second, it can be difficult to separate aboveground from belowground biomass. This source of error could particularly impact moss biomass measurements since the transition can be difficult to discern, though can also affect vascular plant biomass measurements if belowground rhizomes that form shoot tissue are excluded. Third, if plants are highly intermixed, it can be difficult to cleanly separate aboveground biomass into taxonomic or functional groups. Lichens are particularly prone to underestimation because small filamentous lichens are difficult to separate from litter, and crustose lichens were not harvested. Fourth, since it was not feasible to harvest trees and tall shrubs on sample plots, their aboveground biomass was instead estimated using stem diameter measurements and allometric models. Individual research teams selected and applied the allometric models they deemed most suitable, though it is important to acknowledge the dearth of allometric models for most Arctic trees and shrubs. In total, about 4.4% of plant biomass measurements in the synthesis dataset were derived using this approach and are thus subject to allometric model uncertainty. Efforts to reduce measurement uncertainty and improve data quality could focus on developing new allometric models for Arctic trees and shrubs, as well as establishing good-practice guidelines for measuring plant aboveground biomass in Arctic ecosystems.

The synthesis dataset has a slight taxonomic bias towards vascular plants over non-vascular plants. Specifically, herb, shrub, and tree biomass were measured on 89–97% of sample plots, but lichen and bryophyte biomass were measured on 67–72% of sample plots. This is likely due to greater research emphasis on vascular plants and, as discussed above, challenges with measuring lichen and bryophyte biomass. We encourage researchers to measure biomass for every plant functional type found on their sample plots whenever possible.

The geolocation accuracy of the field measurements should be considered when using the synthesis dataset for geospatial analyses. For each dataset, we assembled the best available coordinates, resulting in plot-level and site-level coordinates for 72% and 28% of measurements, respectively. Additionally, plot and site coordinates were determined using a variety of GPS units, with accuracies ranging from <1 meter to tens of meters. If necessary, users can filter the biomass measurements by coordinate type (i.e., *plot* or *site*), though we caution that not all plot-level coordinates may be suitable for geospatial analyses that require meter or submeter accuracy.

The synthesis dataset includes plant biomass measurements from across the Arctic (e.g., Figs. 1, 2a,b); however, there are geographic biases and gaps in data coverage. The distribution of sample plots was biased towards northern Europe (33%) and Alaska, USA (25%), with much lower density of sample plots across Canada (20%), Russia (16%) and Greenland (6%). Regions with data gaps include large parts of northern Canada, the Taimyr Peninsula and Chukchi Peninsula in Russia, and most of Greenland. These general regions have been identified as under sampled in prior assessments of geographic sampling biases in Arctic terrestrial research^63–65^. Regional and bioclimatic biases and gaps in existing field measurements of plant biomass could be quantitatively assessed using the synthesis dataset, which could help strategically prioritize future efforts to measure and monitor ecological changes occurring in the Arctic.

The time periods represented by the synthesis dataset should also be considered. Plant biomass was measured on sample plots between June and early September from 1998 to 2022 (Fig. 2c,d), with about two thirds of sample plots measured after mid-July. In tundra ecosystems, total plant aboveground biomass tends to reach a summer maximum between mid-July and late-August depending on growing season conditions, vegetation composition, and herbivory^66–68^. We estimate that plant biomass measurements made after mid-July are likely within ±15% of the summer maximum based on seasonal changes in plant aboveground biomass measured on sample plots in the Oroarctic^68^, Low Arctic^67^, and High Arctic^66^. Plant biomass measurements made before mid-July likely underestimate the summer maximum to a greater degree. When using the synthesis dataset, plant biomass measurements can be temporally filtered to fit the research needs.

We included as many individual datasets across the Arctic as possible within time limits allocated to this work but acknowledge the synthesis does not include all existing datasets. We prioritized datasets from observational studies carried out in the 21^st^ century where plant biomass was separately measured for every functional type and where sample plots were accurately geolocated. In some cases, it was not possible to obtain access to datasets, or incorporate datasets that very recently became available^69,70^. We programmatically created the synthesis dataset using custom R scripts, and thus the synthesis dataset could in the future be updated to include additional datasets and other refinements.

*The Arctic plant aboveground biomass synthesis dataset* can be used for a variety of ecological applications that include monitoring, mapping, and modeling spatial patterns and temporal changes in plant biomass. Sample plots in the synthesis dataset could serve as ecological baselines for long-term monitoring and experimental manipulations (e.g., warming chambers, herbivore exclosures), or used to analyze geographic biases and gaps in existing field data^63,64^. These field data could be linked with satellite or airborne observations to create maps of plant biomass that can used for carbon accounting^71^, land use planning^29^, terrestrial ecosystem model evaluation^72^, and other ecological applications^5,39^. These field data can also be directly used to evaluate and improve terrestrial ecosystem models and their simulations of Arctic ecosystem response to climate warming^36,73–75^. Overall, *The Arctic plant aboveground biomass synthesis dataset* is a unique dataset suitable for many ecological applications.

## Data Availability

Datasets were harmonized, summarized, and visualized using custom scripts written in R (version 4.0). These scripts are publicly available on GitHub (https://github.com/logan-berner/arctic_plant_biomass_synthesis_scripts) and archived on Zenodo^76^.
